# Investigating volumetric repainting to mitigate interplay effect on 4D robustly optimized lung cancer plans in pencil beam scanning proton therapy

**DOI:** 10.1002/acm2.13183

**Published:** 2021-02-18

**Authors:** Suresh Rana, Anatoly B. Rosenfeld

**Affiliations:** ^1^ Department of Medical Physics The Oklahoma Proton Center Oklahoma City Oklahoma USA; ^2^ Department of Radiation Oncology Miami Cancer Institute Baptist Health South Florida Miami FL USA; ^3^ Department of Radiation Oncology Herbert Wertheim College of Medicine Florida International University Miami FL USA; ^4^ Centre for Medical Radiation Physics (CMRP) University of Wollongong Wollongong NSW Australia

**Keywords:** 4D robust optimization, interplay effect, lung cancer, Monte Carlo, pencil beam scanning

## Abstract

**Purpose:**

The interplay effect between dynamic pencil proton beams and motion of the lung tumor presents a challenge in treating lung cancer patients in pencil beam scanning (PBS) proton therapy. The main purpose of the current study was to investigate the interplay effect on the volumetric repainting lung plans with beam delivery in alternating order (“down” and “up” directions), and explore the number of volumetric repaintings needed to achieve acceptable lung cancer PBS proton plan.

**Method:**

The current retrospective study included ten lung cancer patients. The total dose prescription to the clinical target volume (CTV) was 70 Gy(RBE) with a fractional dose of 2 Gy(RBE). All treatment plans were robustly optimized on all ten phases in the 4DCT data set. The Monte Carlo algorithm was used for the 4D robust optimization, as well as for the final dose calculation. The interplay effect was evaluated for both the nominal (i.e., without repainting) as well as volumetric repainting plans. The interplay evaluation was carried out for each of the ten different phases as the starting phases. Several dosimetric metrics were included to evaluate the worst‐case scenario (WCS) and bandwidth based on the results obtained from treatment delivery starting in ten different breathing phases.

**Results:**

The number of repaintings needed to meet the criteria 1 (CR1) of target coverage (D_95%_ ≥ 98% and D_99%_ ≥ 97%) ranged from 2 to 10. The number of repaintings needed to meet the CR1 of maximum dose (ΔD_1%_ < 1.5%) ranged from 2 to 7. Similarly, the number of repaintings needed to meet CR1 of homogeneity index (ΔHI < 0.03) ranged from 3 to 10. For the target coverage region, the number of repaintings needed to meet CR1 of bandwidth (<100 cGy) ranged from 3 to 10, whereas for the high‐dose region, the number of repaintings needed to meet CR1 of bandwidth (<100 cGy) ranged from 1 to 7. Based on the overall plan evaluation criteria proposed in the current study, acceptable plans were achieved for nine patients, whereas one patient had acceptable plan with a minor deviation.

**Conclusion:**

The number of repaintings required to mitigate the interplay effect in PBS lung cancer (tumor motion < 15 mm) was found to be highly patient dependent. For the volumetric repainting with an alternating order, a patient‐specific interplay evaluation strategy must be adopted. Determining the optimal number of repaintings based on the bandwidth and WCS approach could mitigate the interplay effect in PBS lung cancer treatment.

## 1 Introduction

Lung cancer treatment using pencil beam scanning (PBS) proton therapy presents two major challenges. First, the proton beam needs to transverse inhomogeneities, and the accuracy of the proton dose calculation algorithm in predicting the dose in the lung becomes paramount. Published literature has reported that the analytical pencil beam algorithms over‐estimate the dose in the lung.^1^ Monte Carlo dose calculation engines are becoming available in the commercial treatment planning systems (TPSs). Researchers are advocating the use of the Monte Carlo for dose calculations if the proton beam encounters low‐ and high‐density interfaces in its path, such as in the case of lung cancer treatment,^1^, ^2^, ^3^, ^4^ as well as if the proton beam traverses a range shifter, which creates an air gap between the distal end of the range shifter and patient body.^5^, ^6^, ^7^ The second challenge in treating lung cancer with the PBS proton beam is the interplay effect between dynamic pencil proton beams and motion of the lung tumor.^8^, ^9^, ^10^, ^11^, ^12^, ^13^, ^14^, ^15^, ^16^, ^17^, ^18^, ^19^ To mitigate the interplay effect in proton therapy, several strategies have been proposed. These strategies include breath‐hold, abdominal compression, gating, and repainting.^8^, ^12^, ^20^

Repainting (also referred to as rescanning) allows the energy layers of the proton beam to be delivered more than once to achieve statistical averaging of motion effects.^12^, ^17^, ^18^ Volumetric repainting is delivered by repetitive scanning through the whole target volume, whereas in layered repainting, the energy layer is rescanned more than once before switching to the next energy layer.^12^, ^17^, ^18^ A benefit of volumetric and layered repainting over gating and breath‐hold is the lack of external equipment that could require patient cooperation.^19^ Several studies^4^, ^12^, ^14^, ^17^ have investigated the potential use of volumetric repainting in lung cancer and compared the volumetric repainting against layered repainting, providing contradictory conclusions. For instance, Seco et al.^17^ showed that the volumetric repainting produced better results than layered repainting, whereas Grassberger et al.^14^ showed that layered repainting is superior or equal to volumetric repainting. Engwall et al.^12^ found that offline breath‐sample layered repainting is superior to simple layer repainting and volumetric repainting. In offline breath‐sample layered repainting strategy, the layer rescans for each energy level are spread uniformly over the breathing cycle.^12^ Recently, Wang et al.^4^ reported that a total of four volumetric repaintings were found to be optimal on ProteusPLUS proton system (Ion Beam Applications, Louvain‐la‐Neuve, Belgium) when they examined it on a moving anthropomorphic lung phantom. Wang et al.^4^ demonstrated the feasibility of delivering volumetric repainting plans in a clinical setting.

**FIG. 1:**
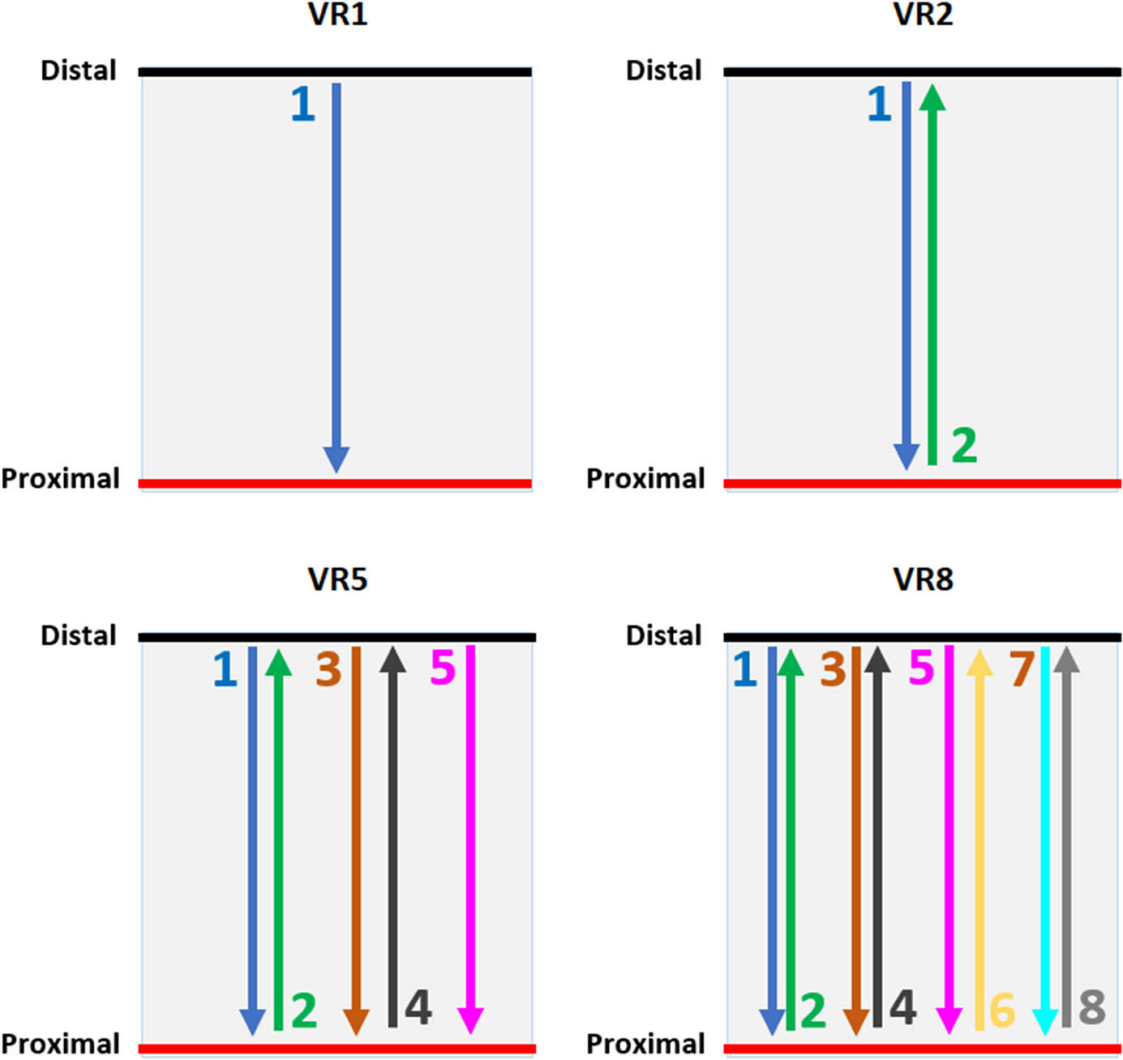
Examples of beam delivery directions in nominal VR1 (no repainting) and volumetric repainting plans with an alternating order; VR2 = 2 repaintings, VR5 = 5 repaintings, VR8 = 8 repaintings; Note: beam delivery starts from the distal energy layer to the proximal energy layer, and then follows an alternating order.

To take advantage of the volumetric repainting technique in mitigating interplay in lung cancer, the proton delivery system needs to have a faster layer switching mechanism.^18^, ^19^ However, the volumetric repainting technique is manufacturer specific, and volumetric repainting capability may vary among different proton machines from the same manufacturer.^19^ The above‐mentioned volumetric repainting studies^4^, ^12^, ^14^, ^17^ on the lung cancer were conducted with beam delivery sequence in “down” direction only such that the proton beam is delivered from the deepest layer (highest energy) to the most proximal layer (lowest energy), and then scans are repeated (i.e., from the highest energy to the lowest energy). Figure 1 At Miami Cancer Institute, ProteusPLUS proton therapy system with a PBS dedicated nozzle is employed.^21^, ^22^ Recently, in an effort to decrease the layer switching time, a magnetic field regulation feature has been implemented on the proton delivery system.^23^, ^24^ For magnetic field regulation mode, Hall probes are mounted inside specific groups of magnets in the beamline. This allows the reduction in beam stabilization delays and layer switching time in both “down” and “up” directions.^23^, ^24^ The “up” direction means the proton beam is delivered from the most proximal layer (lowest energy) to the distal layer (highest energy). Figure 1 The use of magnetic field regulation has decreased the layer switching time to ~ 0.9 s in the “down” direction and ~ 1.3 s in the “up” direction.^23^, ^24^ This provides the feasibility of delivering volumetric repainting using an alternating order with beam delivery sequences in “down” and “up” directions as shown in Fig. 1.

The availability of faster energy layer switching in PBS proton therapy has generated a renewed interest in utilizing volumetric repainting in a clinical environment. In the current study, the authors aim to investigate the interplay effect on the volumetric repainting lung plans that are generated using an alternating order (“down” and “up” directions), and explore the number of volumetric repaintings needed to achieve acceptable lung cancer PBS proton treatment plan. For a volumetric repainting plan with beam delivery sequence in “down” direction only, the beamline needs to be switched from the lowest energy to the highest energy of the given treatment field when scans are repeated in depth. Such a delivery technique with big energy steps in magnetic field regulation mode may lead to destabilization of the magnets. It has been reported that big energy steps (of the order of the full energy range) can cause the beam positioning displacements of 1 to 3 mm.^25^ A faster layer switching time in both “up” and “down” directions in magnetic field regulation mode provides the choice in terms of delivering a volumetric repainting plan, that is, repainting in both “up” and “down” directions. To date, previous volumetric repainting studies^4^, ^12^, ^14^, ^17^ on the lung cancer utilized beam delivery in “down” direction only. In the current study, the authors investigated the volumetric repainting technique with an alternating order (“up” and “down” directions) with a focus on several key items that are relevant for its clinical implementation: (i) the interplay effect evaluation on 4D robustly optimized volumetric repainting plans with an alternating order, (ii) the worst‐case scenario (WCS) evaluation based on ten different breathing phases from 4D computed tomography (4DCT) as the starting phases, and (iii) a method to determine the number of volumetric repaintings needed for an acceptable PBS lung cancer treatment plan.

## 2 Methods and Materials

### 2.A Patient cohort

The current retrospective study includes ten lung cancer patients. The selection of the patients was made based on the following criteria:

- 4DCT data set includes all ten phases.
- Tumor motion is greater than 3 mm but less than 15 mm.
- Tumor is not attached to the mediastinum.
- Clinical target volume (CTV) is less than 200 cc.

**FIG. 2:**
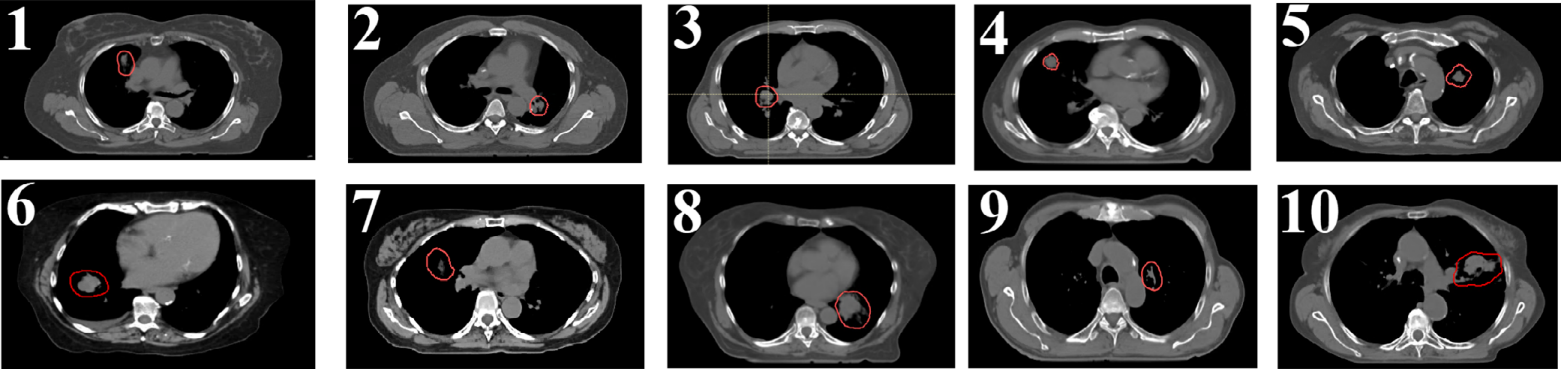
The location of the CTV (red contour) in ten lung patients in the current study. The CTV ranged from 22.10 cc to 181.03 cc, whereas the tumor motion ranged from 3.8 mm to 13.2 mm

The location of the CTV in all ten patients is provided in Fig. 2. The dimension of the CTV ranged from 22.10 cc to 181.03 cc. The tumor motion ranged from 3.8 mm to 13.2 mm. The overall tumor motion was calculated from the magnitude of a 3D vector in the left–right (LR), anterior–posterior (AP), and superior–inferior (SI) directions.

### 2.B Contouring, registration, and treatment planning

In this retrospective study, gross tumor volume (GTV) was contoured in all ten phases of the 4DCT. The CTV was then generated by an isotropic margin of 5 mm around the GTV in all ten phases. Deformable registration was performed between the average intensity projection CT and ten phases from the 4D CT data set using ANAtomically CONstrained Deformation Algorithm (ANACONDA) within RayStation TPS.^11^

In the current study, the total dose prescription to the CTV was 70 Gy(RBE) with a fractional dose of 2 Gy(RBE). PBS plans were generated in RayStation TPS (v9B; RaySearch Laboratories, Stockholm, Sweden) using the beam model of an IBA ProteusPLUS PBS machine that has an in‐air one sigma spot size of 3 mm (at the isocenter) for the highest energy of 226.5 MeV.^26^, ^27^ All treatment plans were robustly optimized (patient setup uncertainty = 5 mm; range uncertainty = 3.5%) on all ten phases in the 4D data set. Specifically, 4D robust optimization^11^, ^28^ was performed with the goal of 99% of the CTV receiving at least 99% of the prescription dose. The organs at risk (OARS) such as the heart, spinal cord, normal lung, and esophagus were included in the 4D optimization. The robust objective was applied to the CTV only. All treatment plans were based on the single field uniform dose (SFUD) technique utilizing two to three proton fields. The layer spacing was set by default in RayStation using automatic with scale 1. The layer spacing is calculated based on the Bragg peak width between the proximal 80% and distal 80% of each layer.^29^ The spot spacing was also set by default in using automatic with scale 1. The spot spacing varies as a function of depth.^29^ The Monte Carlo algorithm was used for the 4D robust optimization (10,000 ions/spot), as well as for the final dose calculation (statistical uncertainty of 0.5%) with a grid size of 3 mm. Treatment plans were then normalized, such that 99% of the CTV received 6930 cGy(RBE). These plans are referred to as nominal plans (VR1) with beam delivery in “down” direction only.

### 2.C Volumetric repainting

For each patient, the VR1 plan is used to generate volumetric repainting plans with an alternating order, as shown in Fig. 1. The scripting environment within RayStation was utilized to generate the volumetric repainting plans. Engwall et al.^11^, ^12^ has detailed the method to generate the volumetric repainting plans using a script in RayStation TPS. A minimum monitor unit (MU) of 0.015 as the spot weight was applied for all volumetric repainting plans to ensure the deliverability of the spots on the machine. If the alternating order includes X paintings, the plan is denoted as a VRX plan. For instance, the plan with five paintings with an alternating order is denoted as a VR5 plan.

### 2.D Interplay effect

The interplay effect study was also performed within the RayStation scripting environment.^11^, ^12^ The interplay effect was evaluated for both the nominal VR1 plan (i.e., without repainting) as well as volumetric repainting plans (VRX) that have X paintings in alternating order. For the time structure of the proton beam delivery on the machine, the following parameters were used: motion speed between spots = 250 cm/s; spot delivery time = 4.0 ms/MU; minimum spot weight of the machine = 0.015 MU; energy layer switching time = 1.0 s. For more details on the interplay evaluation process, the readers are recommended to refer to the publications by Engwall et al.^11^, ^12^ and Pfeiler et al.^30^ The interplay evaluation was carried out for each of the ten different phases as the starting phases.

### 2.E Worst‐case scenario analysis

For each treatment plan (VR1 and VRX) of a given patient, the results were obtained for each phase including the starting phase. The following metrics were used to evaluate the WCS values and DVH bandwidths from the results of treatment delivery starting in ten different phases.

- Target coverage: D_95%_ and D_99%_.
- Hot spot: D_1%_
- Homogeneity Index: D_99%_/D_1%_
- DVH bandwidths

### 2.F Criteria for acceptable plan

Currently, there is no consensus on acceptable interplay effect evaluation criteria for lung proton therapy. The acceptance criteria used in the current study are provided in Table 1. If a given treatment plan with X number of repaintings met the criteria 1 (CR1) of all seven metrics, it was considered “acceptable.” However, if X number of repaintings did not meet the CR1 of all seven metrics, the number of repaintings was increased until CR1 of each metric was fulfilled. The maximum allowable repainting was set to 10. If a final plan met CR1 of at least five metrics but met criteria 2 (CR2) of all metrics, it was considered “acceptable with a minor deviation.”

(1) $$\Delta D_{1\%}=\frac{\left(D_{1\%}^{\mathrm{WCS}}‐D_{1\%}^{\mathrm{Nominal}}\right)}{D_{1\%}^{\mathrm{Nominal}}}\times 100$$

(2) $$\Delta HI=({\mathrm{HI}}^{\mathrm{Nominal}}‐{\mathrm{HI}}^{\mathrm{WCS}})$$

(3) $${\Delta D}_{\mathrm{avg}}=\frac{\left(\Delta D_{95\%}+\Delta D_{96\%}+\Delta D_{97\%}+\Delta D_{98\%}+\Delta D_{99\%}\right)}{5}$$

(4) $$\Delta D_{X\%}=\frac{\left(D_{X\%}^{\mathrm{Nominal}}‐D_{X\%}^{\mathrm{WCS}}\right)}{D_{\mathrm{Rx}}}\times 100$$

where, D_Rx_ is prescription dose; and X = 95%, 96%, 97%, 98%, and 99%.

$${\mathrm{BW}}_{\mathrm{avg}}\;for\;target\;coverage=$$

(5) $$\frac{\left(BW\;at\;D_{95\%}+BW\;at\;D_{96\%}+BW\;at\;D_{97\%}+BW\;at\;D_{98\%}+BW\;at\;D_{99\%}\right)}{5}$$

where, BW = bandwidth of all ten scenarios from the DVHs at dose to the X% of the CTV; X = 95%, 96%, 97%, 98%, and 99%.

$$\mathrm{DVH}\;{\mathrm{BW}}_{\mathrm{avg}}\;for\;high\;Dose=$$

(6) $$\frac{\left(BW\;at\;D_{1\%}+BW\;at\;D_{2\%}+BW\;at\;D_{3\%}+BW\;at\;D_{4\%}+BW\;at\;D_{5\%}\right)}{5}$$

where, BW = bandwidth of all ten scenarios from the DVHs at dose to the X% of the CTV; X = 1%, 2%, 3%, 4%, and 5%.

**TABLE 1:** Metrics to evaluate the WCS and bandwidth based on the results obtained from treatment delivery starting in ten different breathing phases. Each phase is considered as one scenario.

Metric	Criteria 1 (CR1)	Criteria 2 (CR2)
D_95%_	≥98% (6860 cGy(RBE))	97%≤ × < 98%
D_99%_	≥97% (6790 cGy(RBE))	96%≤ × < 97%
∆D_1%_	<1.5%	1.5%≤ × < 2.5%
∆D_avg_.	<2%	2%≤x < 2.5%
∆HI	<0.030	0.030 < × <0.040
BW_avg._ for target coverage	<100 cGy	<150 cGy
BW_avg._ in high‐dose region	<100 cGy	<150 cGy

## 3 Results

### 3.A Target coverage

**FIG. 3:**
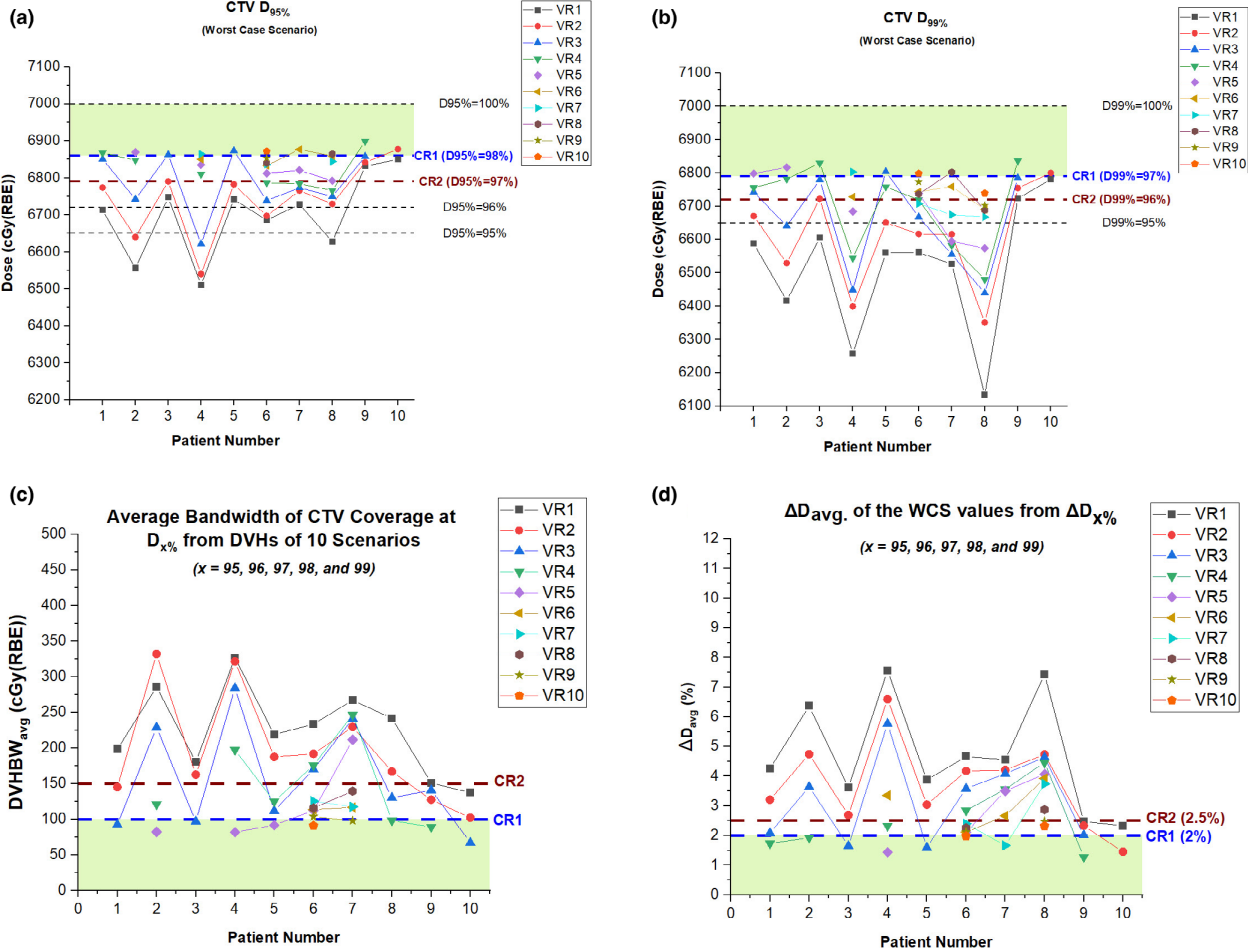
The WCS CTV (a) D_95%_, (b) D_99%_, (c) DVHBW_avg_., and (d) ∆D_avg_. for the target coverage in ten patients. Note: CR1 = Criteria 1, CR2 = Criteria 2; VR1 = Nominal plan with no repaintings, VRX = Volumetric repainting with an alternating order where X is the number of repaintings.

Figures 3(a) and 3(b) show the WCS results for the CTV D_95%_ and D_99%_, respectively. The maximum number of repaintings needed to meet the target coverage varied among patients. The number of repaintings needed to meet the CR1 (D_95%_ ≥ 98% and D_99%_ ≥ 97%) ranged from 2 to 10. Another observation made for the target coverage was that if X number of repaintings meets the CR1 for D_95%_, the same number of repaintings may not always meet the CR1 for D_99%_. For instance, in patient 1, four repaintings were not sufficient to meet the CR1 for D_99%_ but were able to satisfy the CR1 for D_95%_. Figure 3(d) shows the WCS results for the CTV ∆D_avg_. The number of repaintings needed to meet the CR1 (∆D_avg_ < 2%) ranged from 2 to 10. One patient (#8) did not meet the CR1 of D_99%_ and ∆D_avg_. but met the CR2 of these metrics.

### 3.B Homogeneity and hot spot

**FIG. 4:**
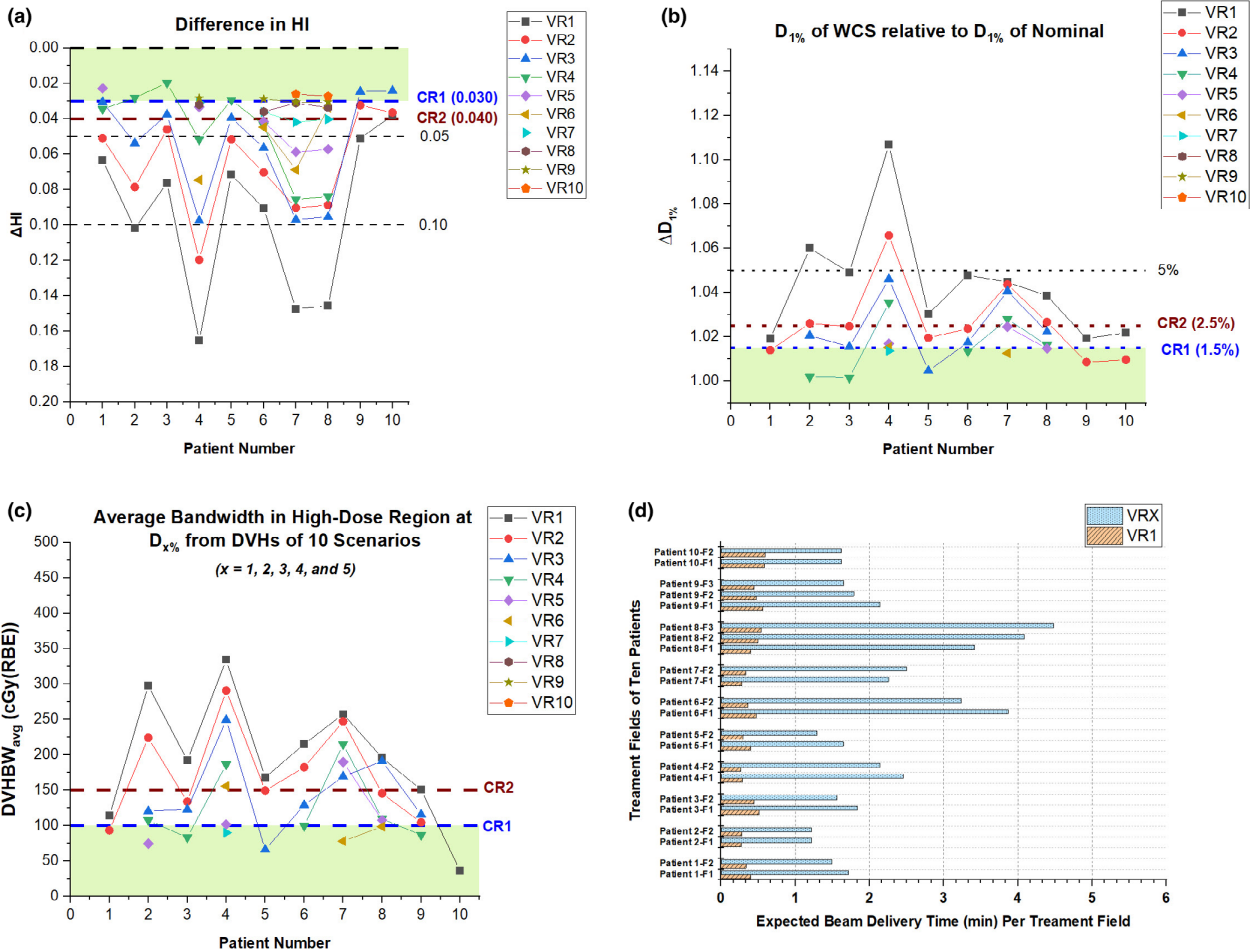
The WCS of CTV (a) ∆HI, (b) ∆D_1%_, (c) DVHBW_avg_.in high‐dose region, and (d) expected beam delivery time per treatment field in the nominal plan (VR1) and selected volumetric repainting plans (VRX) with an optimal number (X) of repaintings in alternating order. The selection of VRX plans is illustrated in Fig. 4. Note: CR1 = Criteria 1, CR2 = Criteria 2; VR1 = Nominal plan with no repaintings, VRX = Volumetric repainting with an alternating order where X is the number of repaintings.

Figure 4(a) shows the WCS results for the CTV ∆HI. To meet the CR1 (∆HI < 0.03), the following observations were made: three repaintings in patients #9 and #10, four repaintings in patients #1, #2, #3, and #5, nine repaintings in patients #4 and #6, and ten repaintings in patients #7 and #8. Figure 4(b) shows the WCS results for the CTV ∆D_1%_. The observation for ∆D_1%_ was similar to the one for ∆HI. The number of repaintings needed to meet the CR1 (∆D_1%_ < 1.5%) ranged from 2 to 7. Two out of ten patients needed more than five repaintings to achieve ∆D_1%_ < 1.5%.

### 3.C DVH Bandwidth

The evaluation of the average DVH bandwidth (BW_avg_.) is illustrated in [Figs. 3(c) and 4(c)]. As shown in [Eqs. (5) and (6)], the BW at a given dosimetric parameter was obtained by calculating the widths of DVHs from ten scenarios. For the target coverage region [Fig. 3(c)], the number of repaintings needed to meet CR1 (<100 cGy) ranged from 3 to 10. For the high‐dose region [Fig. 4(c)], the number of repaintings needed to meet CR1 (<100 cGy) ranged from 1 to 7.

### 3.D Overall plan evaluation

**FIG. 5:**
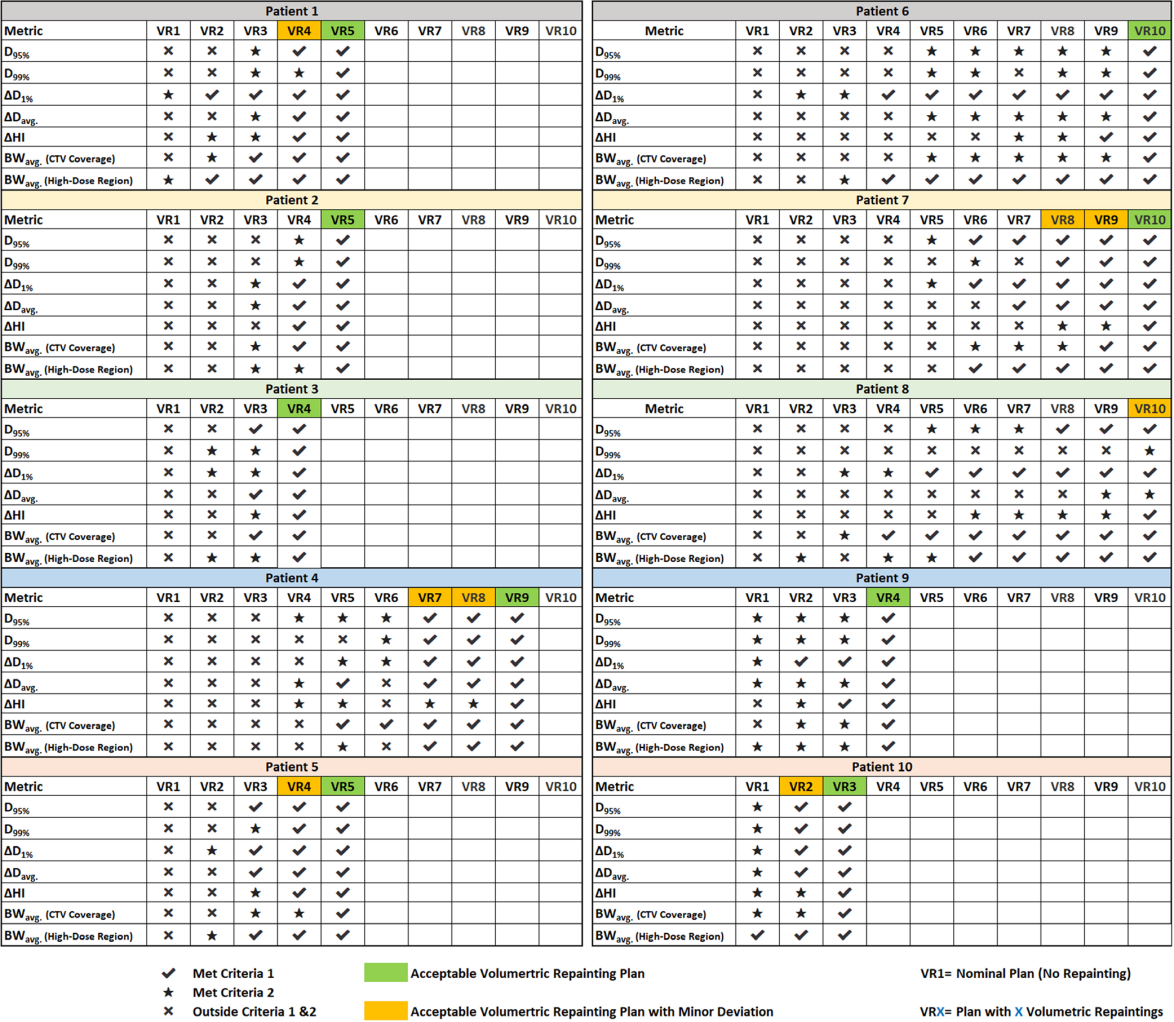
A chart displaying a selection of an optimal number of repaintings based on the criteria described in section 2.6; VR1 = Nominal plan with no repaintings, VRX = Volumetric repainting with an alternating order where X is the number of repaintings.

**FIG. 6:**
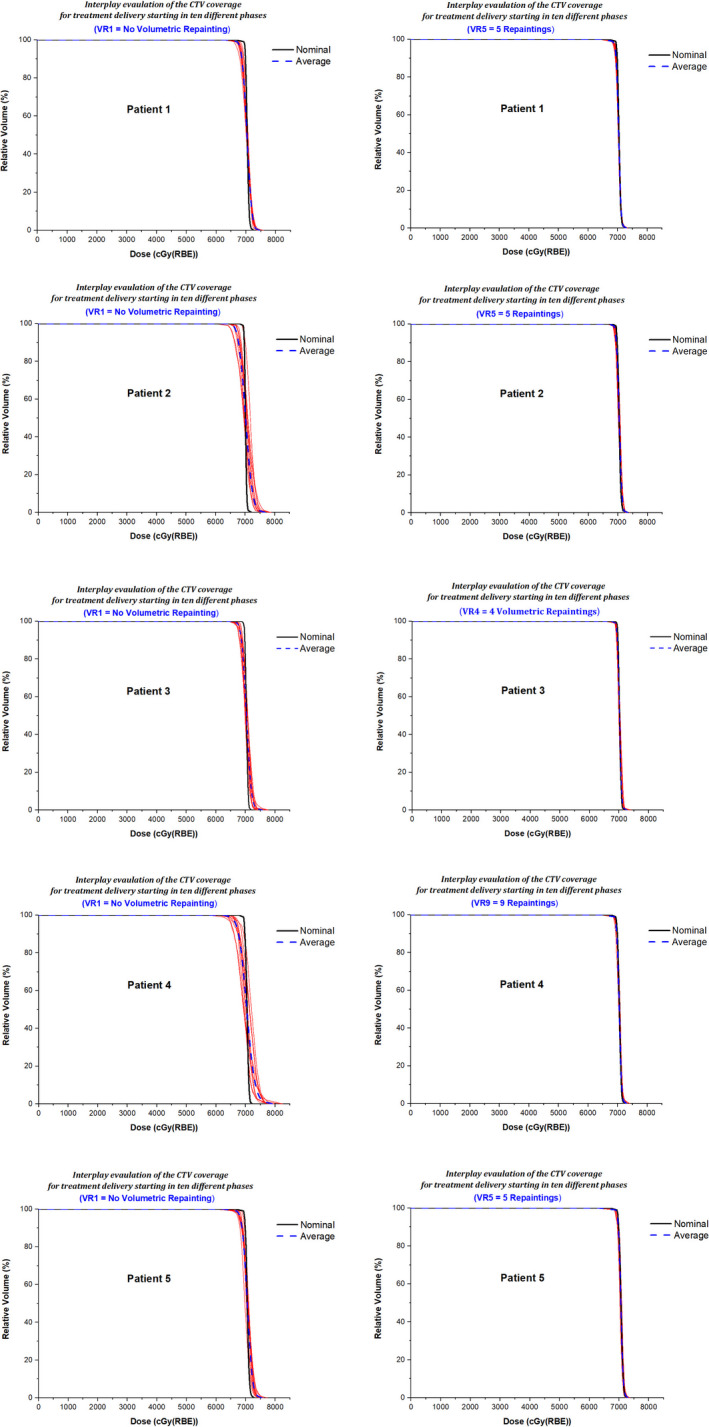
Patients 1–5; Interplay DVHs (red lines) for treatment delivery starting in ten different phases, an average of interplay DVHs (blue dashed line), and nominal DVH (black line). The left panel displays the results for the nominal plan (VR1) without repaintings, and the right panel shows the results for the selected volumetric repainting plans (VRX) with an optimal number of repaintings.

**FIG. 7:**
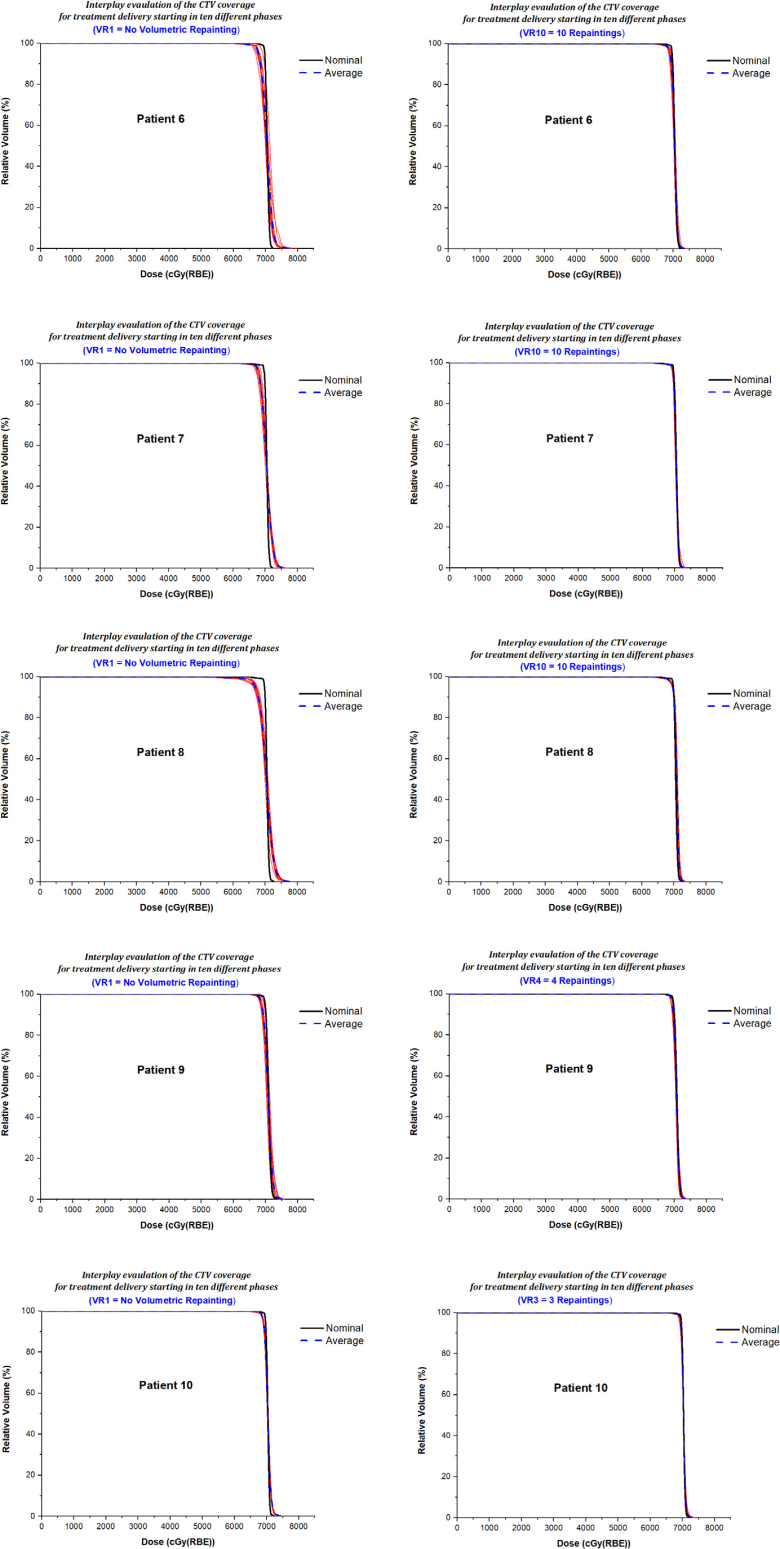
Patients 6–10; Interplay DVHs (red lines) for treatment delivery starting in ten different phases, an average of interplay DVHs (blue dashed line), and nominal DVH (black line). The left panel displays the results for the nominal plan (VR1) without repaintings, and the right panel shows the results for the selected volumetric repainting plans (VRX) with an optimal number of repaintings.

Based on the criteria described in section 2.6, the final plan evaluation showed that acceptable plans were achieved for nine patients, whereas one patient had an acceptable plan with a minor deviation. Figure 5 shows the chart of each patient displaying if the metric has met CR1 and CR2. Figures 6 and 7 illustrate the interplay DVHs for treatment delivery starting in ten different phases, an average of interplay DVHs, and nominal DVH in all ten patients.

- For patient #10 (CTV = 103.92 cc; tumor motion = 4.7 mm), three repaintings were sufficient to achieve acceptable plans.
- For patients #3 (CTV = 26.46 cc; tumor motion = 8.1 mm) and #9 (CTV = 63.26 cc; tumor motion = 3.8 mm), four repaintings were sufficient to achieve acceptable plans.
- For patients #1 (CTV = 36.45 cc; tumor motion = 7.2 mm), #2 (CTV = 34.22 cc; tumor motion = 5.8 mm), and #5 (CTV = 24.37 cc; tumor motion = 4.8 mm), five repaintings were sufficient to achieve acceptable plans.
- For patient #4 (CTV = 26.05 cc; tumor motion = 10.1 mm), nine repaintings were sufficient to achieve acceptable plans.
- For patients #6 (CTV = 39.25 cc; tumor motion = 10.2 mm) and #7 (CTV = 22.10 cc; tumor motion = 8.8 mm), ten repaintings were sufficient to achieve an acceptable plan.
- For patient #8 (CTV = 181.03 cc; tumor motion = 13.2 mm), ten repaintings were needed to achieve acceptable plan with a minor deviation.

### 3.E Layers and Spots

For nominal plans (VR1), the number of layers per beam ranged from 13 to 28, whereas the total spots per beam ranged from 598 to 2150. For acceptable plans, the number of layers per beam ranged from 61 to 213, and the number of spots per beam ranged from 2519 to 4886. For an acceptable plan with minor deviation, the number of layers per beam ranged from 171 to 233, and the number of spots per beam ranged from 5961 to 6490.

### 3.F Treatment Delivery Time

The expected treatment delivery times for the nominal plans without repainting (VR1) and selected volumetric repainting plans (VRX) with an optimal number of repaintings of each patient are provided in [Figure 4(d)]. For acceptable plans, treatment delivery time per beam varied from 1.22 min (VR5 in patient #2) to 3.87 min (VR10 in patient #6). For an acceptable plan with minor deviation, treatment delivery time per beam varied from 3.42 min (VR10 in patient #4) to 4.49 min (VR10 in patient #8).

## 4 Discussion

The current study evaluated the interplay effect of 4D robustly optimized volumetric repainting lung cancer plans. We have presented the volumetric repainting technique with an alternating order, which was not explored in previous studies^4^, ^12^, ^14^, ^17^ on lung cancer. Recently, Rana et al.^23^ performed an experimental study quantifying the impact of magnetic field regulation in conjunction with the volumetric repainting technique (alternating order) on the spot positions and range in PBS protons. Rana et al.^23^ demonstrated the feasibility of delivering volumetric repainting QA plans with an alternating order on the clinical proton machine. While the study by Rana et al.^23^ was focused on the machine QA, the current study was performed on the 4DCT data set of lung cancer to investigate the mitigation of interplay effect by using an alternating order in volumetric repainting technique.

The volumetric repainting is not clinically implemented at many proton centers. One of the reasons could be due to longer treatment time because of slower energy layer switching. In the current study, we approximated the layer switching time of 1 s for both “down” and “up” directions based on the findings by Rana et al.,^23^ although layer switching time could slightly vary from 1 s when plans are delivered on the machine. The simulated treatment delivery time for the cohort of patients in the current study showed the feasibility of delivering volumetric repainting plans mitigating the interplay effect. The average time per beam to deliver the acceptable plans, as well as acceptable plans with minor deviation, was 2.2 min (range, 1.2 min–4.5 min). These estimated beam times could slightly vary during the actual delivery of the volumetric repainting plans on the proton machine.

Since the current study was primarily focused on the interplay effect, the robustness of the lung plans was not investigated. However, treatment plans were generated with the objective of achieving clinically acceptable and deliverable robust plans. To achieve this, the current study was performed using a 4D robust optimization feature available in the RayStation TPS. As described in Engwall’s papers,^11^, ^12^ the 4D optimization ensures the entire treatment volume is encompassed in each breathing phase if the proton beam delivery was instantaneous and resulting distortions are purely caused by the interference between the tumor motion and the dynamic proton beam delivery. The addition of robust objective to the CTV with respect to the setup 5 mm and range uncertainties (3.5%) during 4D robust optimization can make treatment plans more robust, especially for the lung tumor volume that has large density variations in the beam path. Such dosimetric benefit comes at the cost of decreasing computational efficiency if robust optimization process includes all ten breathing phases from 4DCT data set.^28^

Currently, there is no consensus in the proton therapy community regarding which metrics can be used to evaluate the interplay effect. In the current study, we included metrics such as D_95%_, D_1%_, and HI. Literature^31^ has shown the importance of achieving homogeneous dose distribution within the target volume. To create homogenous dose distributions in the treatment plan, the current study used the SFUD technique utilizing two to three beams. D_99%_ was included in the evaluation process since this metric could be correlated with the clinical outcome in lung cancer patients.^32^ For the evaluation of these metrics, we utilized the WCS approach based on the results of beam delivery, starting in ten different breathing phases. Each individual phase was considered as one scenario. During the lung cancer treatment, treatment beam delivery could start at any breathing phase, and this can be considered as a random variable. In general, the increase in the number of volumetric repaintings improved the WCS value of given metric and allowed us to mitigate the interplay effect and meet the criteria provided in Table 1. Additionally, we measured the average DVH bandwidths by evaluating dose at several dosimetric parameters that are relevant in the target coverage and high‐dose regions. In general, the DVH bandwidths became tighter with an increase in the number of repaintings. For patients #4 and #8, there was no clear trend showing the increase in repaintings resulting in an improvement in bandwidths after five repaintings.

The results presented in the current study demonstrated that the interplay effect for lung cancer was highly patient dependent. This observation is in alignment with previously published studies on the PBS proton therapy for lung cancer.^8^, ^10^, ^11^, ^12^, ^13^, ^14^, ^15^, ^20^, ^33^ For ten patients in the current study, it was not possible to determine the exact correlation between the number of repaintings vs tumor size, tumor location, and tumor motion. Although a larger tumor motion seems to require a higher number of repaintings in the current study, more data with varying tumor motion are necessary to establish the correlation between the number of repaintings and tumor motion. Hence, for the volumetric repainting with an alternating order, instead of applying a fixed number of repaintings across all lung cancer patients, a patient‐specific interplay evaluation strategy must be adopted. This will yield an optimal number of volumetric repaintings for an individual lung cancer patient.

The current study was focused on the volumetric repainting technique with an alternating order. We did not investigate other repainting strategies such as layer repainting and volumetric repainting technique in the “down” direction only. These are the limitations of our study. One of the goals of the current study was to demonstrate the feasibility of mitigating the interplay effect using a volumetric repainting technique with an alternating order rather than to make a comparison against the layer repainting. The use of layer repainting to mitigate the interplay effect cannot be ignored. In the next study, we will make the direct comparison between the volumetric repainting (with alternating order) and layer repainting by providing the dosimetric and radiobiological results.

On the IBA ProteusPLUS machine, if the current regulation feature is employed, the energy layer switching time in the “up” direction can take up to 6 s.^23^, ^24^ Such a large energy switching time is not clinically acceptable. However, the use of magnetic field regulation feature on the IBA ProteusPLUS machine can reduce the energy layer switching time in the “up” direction from ~ 6 s to ~ 1.3 s.^23^, ^24^ Such a decrease in the energy layer switching time demonstrates the feasibility of delivering the volumetric repainting plan with an alternating order and improve the beam delivery efficiency. Another important point to note is that the current study was performed with an assumption of proton beam delivery using magnetic field regulation on the IBA ProteusPLUS machine. In this scenario, Hall probes in the beamline measure the magnetic field in real time and remove the requirement of cycling of the magnets at each set range in the “up” direction.^23^, ^24^ This has led to a decrease in the energy layer switching time and making it feasible to deliver the field in smaller energy steps in the “up” direction.^23^, ^24^ Pedroni *et al*.^25^ reported up to 3 mm in beam positioning displacements for big energy steps (of the order of the full energy range). In the magnetic field regulation mode, big energy steps in the treatment field may cause destabilization of the magnets and a greater fluctuation in Hall probe readings, thus potentially producing errors in spot positions.^23^ The utilization of the “up” direction in the treatment plan can eliminate the need to switch from the lowest energy (proximal layer) to the highest energy (distal layer) of the given treatment field when volumetric scans are repeated. In the magnetic field regulation mode on the IBA ProteusPLUS machine, we recommend delivering a volumetric repainting plan with smaller energy steps (5 MeV or less). More technical details on the magnetic field and current regulation features and volumetric repainting technique on the IBA ProteusPLUS machine can be found in previous publications.^23^, ^24^

The interplay effect results presented herein are more relevant for an IBA ProteusPLUS PBS machine, which employs magnetic field regulation feature, but not for the current regulation feature. The readers must be aware of the fact that the proton beam delivery systems are machine‐ and manufacturer specific, and performance of repainting can vary among different PBS proton machines.^34^ An independent machine‐specific validation for the repainting techniques can provide more accurate estimations of the interplay effect. We acknowledge that the experimental measurements for the interplay effect were not performed in the current study. In the near future, we aim to perform an experiment using a moving phantom simulating different magnitudes of motion and investigate the computed vs measured doses of repainting plans (layer and volumetric).

At present, to the best of our knowledge, an interplay evaluation module is not currently available in the clinical versions of the proton TPSs. The implementation of interplay evaluation within TPS would not only give us the confidence in using the volumetric repainting technique in the clinical environment but also provides a tool to the clinicians to select the optimal number of repaintings. This can result in homogenous dose distributions and maintain target coverage leading to a better clinical outcome for PBS lung cancer patients.

## 5 Conclusion

The interplay effect was evaluated on the 4D robustly optimized lung cancer plans (tumor motion < 15 mm) for the volumetric repainting technique with an alternating order. The number of repaintings required to mitigate the interplay effect was found to be patient dependent. Determining the optimal number of repaintings based on the bandwidth and WCS approach from DVHs of ten breathing phases could mitigate the interplay effect in PBS lung cancer treatment. It is recommended to perform patient‐specific interplay evaluation for PBS lung cancer plans.

## Conflict of Interest

No conflict of interest.
